# A framework for assessing the impact of pharmaceutical reimbursement policies on incentives to innovate

**DOI:** 10.1186/2052-3211-8-S1-P26

**Published:** 2015-10-05

**Authors:** Elizabeth Docteur, Joshua Cohen, Brian K Bruen, Ruth Lopert, Avi Dor, Chuck Shih

**Affiliations:** 1 Elizabeth Docteur Consulting, Alexandria VA 22314, USA; 2 Tufts University, Tufts Center for the Study of Drug Development, Boston, MA, 02111, USA; 3 George Washington University, Milken Institute of Public Health and Health Services, Department of Health Policy, NW. Washington, DC, 20052, USA; 4 The Pew Charitable Trusts, 901 E Street, NW, Washington, DC, 20004-2008, USA

## Problem statement

The effect of pharmaceutical reimbursement policies on innovation is of interest to policymakers aiming to ensure access to effective medicines and contain costs while supporting development of effective new treatments for unmet health needs. Yet the ways in which reimbursement policies affect incentives to innovate are not well understood.

## Objectives

To assist policymakers in evaluating the impact of current and prospective new policies, we developed a framework for analyzing the impact of pharmaceutical reimbursement policies on incentives to innovate.

## Methods

The authors reviewed the research literature to identify hypothetical channels through which policies stand to influence the incentive to invest in development of innovative pharmaceutical products, and then developed a conceptual model illustrating direct and indirect channels of influence.

## Results

We developed an analytical framework with which to explore the link between reimbursement and investment in research and development (R&D) yielding incremental, substantial, and radical innovations, as well as in novel products that do not offer new therapeutic advantages over existing treatments.

This framework posits that there are three ways that reimbursement policies and practices can affect an innovator's expected return on investment (EROI) directly. The first is by establishing a particular payment level, which in turn affects average sales price in line with the share of the prospective market represented by the payer. The second is by setting a volume of sales at that payment level, as may occur in the case of competitive bidding, for example. The third is by influencing seller costs associated with development, manufacture, or sale.

Reimbursement policies also stand to influence EROI indirectly by establishing different incentives for key actors. These incentives, in turn, affect effective price, volume and, in some cases, seller costs.

## Conclusions

While researchers have investigated the links between EROI, ROI, and R&D in the pharmaceutical industry, there is no empirical evidence to directly connect ROI with innovation. Establishing such a connection would entail looking at technical questions such as the ability of potential innovators to achieve targeted research outcomes.

## Lessons learned and success factors

Validation of our conceptual model and analytical framework will require further research. Furthermore, application of the framework is limited by the availability of data by which to assess the impact on key outcomes. Nevertheless, the framework can be helpful to policymakers faced with making timely decisions about adoption of new policies in the face of incomplete evidence.

## Acknowledgements

The funding source was the U.S. Department of Health and Human Services, Office of the Assistant Secretary for Planning and Evaluation, Washington, DC, USA.

